# Evidence for plasmid-mediated salt tolerance in the human gut microbiome and potential mechanisms

**DOI:** 10.1093/femsec/fiw019

**Published:** 2016-02-04

**Authors:** Eileen Broaders, Ciarán O’Brien, Cormac G.M. Gahan, Julian R. Marchesi

**Affiliations:** 1 Alimentary Pharmabiotic Centre, University College Cork, College Road, Cork, T12 YT20, Ireland; 2 Department of Microbiology, University College Cork, College Road, Cork, T12 YT20, Ireland; 3 School of Pharmacy, University College Cork, Cork, T12 YT20, Ireland; 4 School of Biosciences, Cardiff University, Cardiff CF103AX, UK; 5 Centre for Digestive and Gut Health, Imperial College London, London W2 1NY, UK

**Keywords:** gut, microbiome, mobile genetic elements, osmotolerance

## Abstract

The human gut microbiome is critical to health and wellbeing. It hosts a complex ecosystem comprising a multitude of bacterial species, which contributes functionality that would otherwise be absent from the host. Transient and commensal bacteria in the gut must withstand many stresses. The influence of mobile genetic elements such as plasmids in stress adaptation within the ecosystem is poorly understood. Using a mobilomic approach we found evidence for plasmid-mediated osmotolerance as a phenotype amongst the Proteobacteria in healthy faecal slurries. A transconjugant carrying multiple plasmids acquired from healthy faecal slurry demonstrated increased osmotolerance in the presence of metal salts, particularly potassium chloride, which was not evident in the recipient. Pyrosequencing and analysis of the total plasmid DNA demonstrated the presence of plasmid-borne osmotolerance systems (including KdpD and H-NS) which may be linked to the observed phenotype. This is the first report of a transferable osmotolerance phenotype in gut commensals and may have implications for the transfer of osmotolerance in other niches.

## Abstract (precis)

There is evidence of a transferable osmotolerance phenotype in gut commensals which may have implications for the transfer of osmotolerance in other niches.

## INTRODUCTION

Osmotic shift is an ecological threat encountered by bacteria in many niches, from farm to fork and beyond, with the gut being no exception. Strategies used by bacteria for osmotic adaptation range from the accumulation of ions to the utilization of compatible solutes. Uptake of potassium is a front line response by *Escherichia coli* to osmotic shock and a critical event for osmotic adaptation (Epstein 1986; Sutherland *et al*. 1986; Booth and Higgins 1990). The cell increases potassium intake, feeding K^+^-glutamate formation in order to reduce osmotic stress in the cytoplasm (Lucht and Bremer 1994). Transport proteins such as KefB/KefC (Miller *et al*. 2000), Ktr, Kup (Epstein and Kim 1971), Kdp (Laimins, Rhoads and Epstein 1981) and their signalling pathways are key to this response. On the other hand, as environmental water potential increases, causing water to be pulled from the cell, microbes may accumulate solutes to help balance osmolarity to the external level. These solutes do not adversely affect cell processes and are therefore known as compatible solutes (Brown and Simpson 1972). Cells can source compatible solutes by two means; synthesis, or uptake through transport systems. Some compatible solutes such as proline, glycine betaine and choline, may have a positive effect on growth rate and these types may be referred to as osmoprotectants (Csonka 1989). Indeed, horizontal gene transfer of genes involved in catabolism of compatible solutes has recently been reported (Collins and Deming 2013).

Previous literature suggests a role for plasmids in salt tolerance in marine environments and saline soils. Evidence was presented by Qureshi and Malik in 1990 which suggested transfer of natural plasmid, obtained from a soil isolate, to non-halophillic *E. coli* K12 and a *Klebsiella* strain-mediated salt tolerance to the recipients (Qureshi and Malik 1990). Additionally, in 1991 a plasmid whose copy number fluctuates with NaCl concentration was described by Takeyama and colleagues in the marine isolate *Synechococcus* sp. NKBG 042902 (Takeyama *et al*. 1991). More recently, the copy number of another plasmid, pSH1 in a *Micrococcus* sp., has been shown to increase with salt concentration suggesting a relationship with salt tolerance (Lobova, Zagrebel’nyi and Popova 2005).

The human gut is a complex environment with a significant proportion of the microbial community largely refractory to culturing (Walker *et al*. 2011). In the current study, we isolated plasmids from the gut that confer a significant salt tolerance phenotype to the host. The exogenous-plasmid isolation approach has been used successfully to obtain conjugative and mobilisable plasmids from manure (Smalla *et al*. 2000), soil and river epilithon (Bale, Day and Fry 1988; Dronen, Torsvik and Top 1999). To our knowledge this is the first application of this approach towards an understanding of plasmids in the human GI tract.

## MATERIALS AND METHODS

### Growth of bacterial strains

A spontaneous mutant of *E. coli* K12 HB101 (DSMZ 1607), with stable resistance to rifampicin, was used as the recipient in bi-parental matings. The recipient strain was grown in nutrient broth containing 50 μg ml^−1^ rifampicin at 37°C whilst shaking (150 rpm). Prior to mating experiments, overnight broth cultures of recipients were grown in the absence of rifampicin. Bacterial strains were maintained at –80°C in their respective media broths containing 8% v/v dimethyl sulfoxide (DMSO) (Sigma, Wicklow, Ireland) or 40% v/v glycerol (Sigma, Wicklow, Ireland).

### Bi-parental matings between faecal slurries and recipients

Exogenous bi-parental filter matings were performed by modifying the tri-parental exogenous method described by Hill and co-workers in 1992 (Hill, Weightman and Fry 1992). The donor population was provided by 10 ml of a 10^−2^ fresh faecal slurry dilution. Negative controls involved plating faecal slurries onto PCA (aerobic) supplemented with rifampicin 50 μg ml^−1^. The recipient strain was a rifampicin resistant spontaneous mutant of *E. coli* HB101 (HB101:30). A 100 μl aliquot of the recipient was added to 10 ml faecal dilution (1 ml faecal slurry in 9 ml phosphate buffered saline. The mixture was passed through 0.45 μm filters (Millipore, Cork, Ireland) which were subsequently placed onto PCA (aerobic) plates. Experiments were performed in triplicate and incubated at 37°C overnight. The following day, filter washes were conducted by placing them in 10 ml sterile one-fourth strength broth and gently agitated. Serial dilutions of the filter washes were carried out and 20 μl was spot plated onto PCA 4% w/v NaCl media to screen for plasmid-containing recipients. Plates were incubated at 37°C overnight.

### DNA manipulation

Genomic DNA was extracted using the Gentra Puregene Yeast/Bact. Kit (Qiagen Ltd, Crawley—West Sussex, UK) according to the manufacturer's instructions. Plasmid DNA extractions were carried out by the method of Birboim and Doily (Sambrook 2001).

### Dual Locus Sequencing Typing to verify strain similarities

Dual Locus Sequencing Typing was conducted to verify that plasmid-containing isolates recovered from exogenous matings were the same strain as the rifampicin resistant recipient used in the experiment. The following housekeeping genes for adenylate kinase *adk* (536 bp locus size) and malate dehydrogenase *mdh* (452 bp locus size) were amplified using the primer pairs mdh-P1 5^′^ -ATG AAA GTC GCA GTC CTC GGC GCT GCT GGC GG-3^′^, mdh-P2 5^′^ -TTA ACG AAC TCC TGC CCC AGA GCG ATA TCT TTC TT-3^′^ and adk-P1 5^′^ -ATT CTG CTT GGC GCT CCG GG-3^′^ adk-P2 5^′^ -CCG TCA ACT TTC GCG TAT TT-3^′^. Reaction mixtures included 50 ng of chromosomal DNA, 20 pmol of each primer, 200 μmol (10 μl of a 2 mM solution) of the dNTPs, 10 μl of 10 X PCR buffer, 5 units of *Taq* polymerase (Invitrogen, UK) and water to 100 μl. Reaction conditions were as follows, 2 min at 95°C, 30 cycles of 1 min at 95°C, 1 min at annealing temp, 2 min at 72°C followed by 5 min at 72°C (Wirth *et al*. 2006).The PCR products were visualized by agarose gel electrophoresis and cloned into a TOPO cloning vector for sequencing as per manufacturer's instructions (Invitrogen, UK). Sequencing was conducted by GATC in Germany using the M13 forward 5^′^-GTA AAA CGA CGG CCA G-3^′^ and reverse 5^′^-CAG GAA ACA GCT ATG AC-3^′^ sequencing primers. Sequences were processed using Bioedit (Hall 1999) aligned and trimmed using the SeqAlign platform in DNASTAR (DNASTAR Inc., Madison, WI, USA). Consensus sequences obtained from three independently amplified PCR products for each gene of the strains in question were checked for allelic affiliation using the *E. coli* MLST Database at the ERI in University College Cork, Ireland (http://mlst.ucc.ie/mlst/dbs/Ecoli).

### Biolog phenotype microarrays

A single Biolog (TECHNOPATH, Ballina, Ireland) phenotype screen was performed for AR1 and HB101:30 using the PM9 (osmolytes), PM11 (anti-cancer agents), PM12 (anti-cancer agents) and PM13 (anti-cancer agents) microarray plates in order to get an indication of potential phenotypes related to the plasmids in AR1. Cell suspensions of the rifampicin resistant *E. coli* HB101 and the exogenously isolated plasmid-containing version, AR1, were prepared separately according to the manufacturer's instructions. A 100 μl volume was added to each well of the MicroPlate, plates were loaded into a BiologOmniLog^®^plate reader and incubated for 48 h. Kinetic data was collected by the BiologOmniLog instrument and software for analysis. Plates were removed to 4°C. Further investigation of five phenotypes was performed through triplicate growth curve experiments measuring optical density at 595 nm over 24 h in TSB media at 37°C. The five phenotypes investigated further were 2% urea, potassium chloride concentrations of 3%, 4%, 5% and 6%.

### Growth analysis of the host and plasmid-containing *E. coli*

Growth measurements were set up as follows. Pre-cultures were grown from a fresh overnight colony to stationary phase in LB containing 1% w/v NaCl. A 2% v/v inoculum of the overnight culture was used for growth curves. A 1 ml aliquot of each overnight was washed twice in sterile one-fourth Ringers solution with final resuspension in the media for the growth curve. A 200 μl aliquot was added to 10 ml of the growth media to be analysed. For turbidometric measurements through OD 595 nm readings, 200 μl of the suspension was added to wells of a 96-well assay plate and placed in a 96-well plate spectrophotometer (Magellan GENios), with readings taken automatically every hour for 24 h. Tests were performed in triplicate from three separate cultures (biological repeats). The results shown in graphs represent the means, standard deviations and error bars for triplicate experiments. Mean, standard deviations and standard errors were calculated using Microsoft Excel. Statistical significance was determined using students *t*-test where a significant effect was observed when P ≤ 0.05. Sigmaplot software (Systat Software Inc., London, UK) was used to construct graphs based on the data obtained.

### Sequencing analysis plasmid DNA

Plasmid DNA from AR1 was prepared by a Maxiprep kit (Qiagen Ltd, Crawley—West Sussex, UK). The maxiprep plasmid DNA was treated with plasmid safe DNase from Epicentre (Epicentre, Madison, WI, USA) to remove genomic DNA. Pyrosequencing was performed by GATC Biotech using titanium chemistry and the Roche 454 GS FLX sequencing platform. Raw data reads and assembled contigs were further analysed using NCBI BLAST (Altschul *et al*. 1997), CAMERA 2.0 and RAST server (Meyer *et al*. 2008). The data was deposited at the EBI's ENA under study accession number PRJEB7899.

## RESULTS

**Figure 1.**
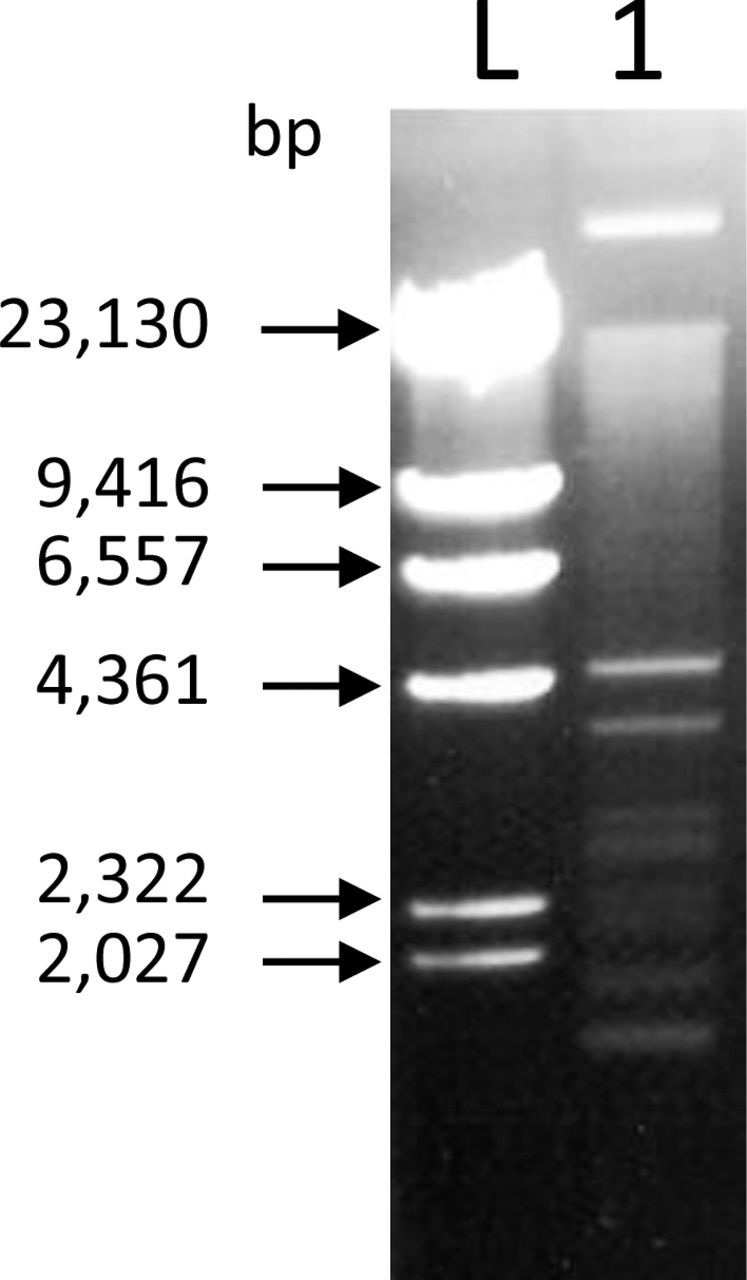
Transconjugant AR Plasmid DNA**.** L is a *Hind*III digest of Lambda (NEB, Ipswich, MA, USA). Lane 1 depicts a 10 μl aliquot of plasmid miniprep from a 5 ml culture of AR1.

Ten colonies were selected from the selective medium, based on colony morphology, and each transconjugant's plasmid profile analysed. Colony morphology of these 10 transconjugants was distinct and larger in size than the rifampicin resistant *E. coli* HB101 recipient. Plasmid preparations revealed multiple bands of plasmid DNA (Fig. 1). All 10 transconjugants showed the same plasmid profile and one transconjugant, named AR1 (anomalous recipient 1), containing multiple plasmids was chosen for subsequent analysis.

### Increased osmotolerance of AR1 in M9 minimal media and NaCl

**Figure 2.**
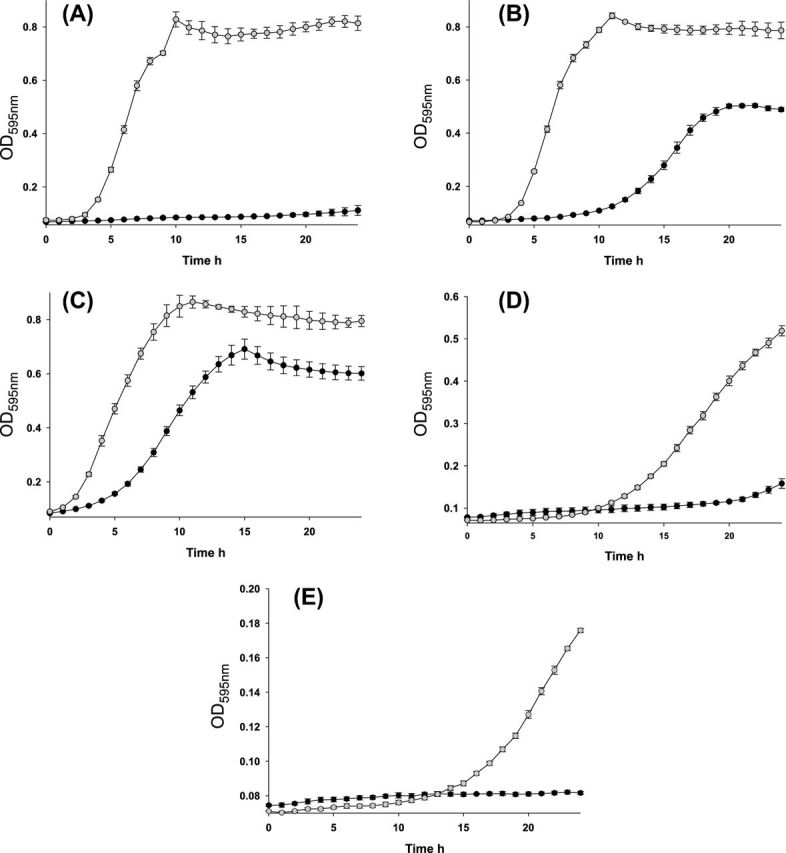
Growth comparisons of the multiplasmid-containing recipient AR1 (filled circle) and the empty recipient HB101:30 (filled circle) in M9 minimal media at 37°C. AR1 (grey circles) grows significantly faster than HB101:30 (black circles) in M9 minimal media containing **(A)** 0.5% w/v NaCl, **(B)** 1% w/v NaCl, **(C)** 3% w/v NaCl, **(D)** 4% w/v NaCl and **(E)** 5% w/v NaCl. Optical density readings were taken at 595 nm. Error bars are standard deviations from the means of data from triplicate experiments. Significance was deemed to be a *P*-value ≤ 0.05.

We initially tested AR1 for characteristics that are known to be plasmid mediated (including antibiotic resistance and resistance to heavy metals); however, no obvious phenotype could be determined. We therefore utilized Biolog phenotypic screens in order to investigate a wider range of conditions. Using this approach we determined a demonstrable increase in the osmotolerance of AR1. Growth comparisons in M9 minimal media provided confirmation that AR1 was fitter than the empty recipient HB101:30 in the presence of sodium chloride. Faster growth was noted for AR1 at all concentrations of NaCl tested in M9 minimal media at 37°C, the most striking being M9 containing 0.5% NaCl where AR1 enters a sharp exponential phase at 4 h and stationary phase at 10 h, but HB101:30 does not reach lag phase (Fig. 2A). At other NaCl concentrations tested AR1 also grew significantly faster than HB101:30. In M9 1% NaCl AR1 begins a sharp exponential phase at 4 h, but HB101:30 experiences an extended lag phase to 10 h and although it does enter stationary phase within the 24 h it does not reach optical density readings comparable to AR1 (Fig. 2B). When grown in M9 3% NaCl both strains have a short lag phase, but AR1 does enter stationary phase sooner and at a higher OD 595 nm reading than HB101:30 (*P* < 0.05; Fig. 2C). A better growth rate is detected for AR1 in M9 4% NaCl also, both strains experience an extended lag phase, but AR1 enters exponential phase at 10 h and reaches an OD 595 nm reading of over 0.5 at 24 h whereas HB101:30 only begins to recover at 21 h and reaches an OD 595 nm of under 0.2 at 24 h. Both strains appear to struggle in M9 5% NaCl with neither exceeding an OD 595 nm reading of 0.2 in the 24 h experiment; however, AR1 does grow from 14 h onward whereas HB101:30 does not for the duration of the curve.

### Increased osmotolerance of AR1 in KCl and urea

**Figure 3.**
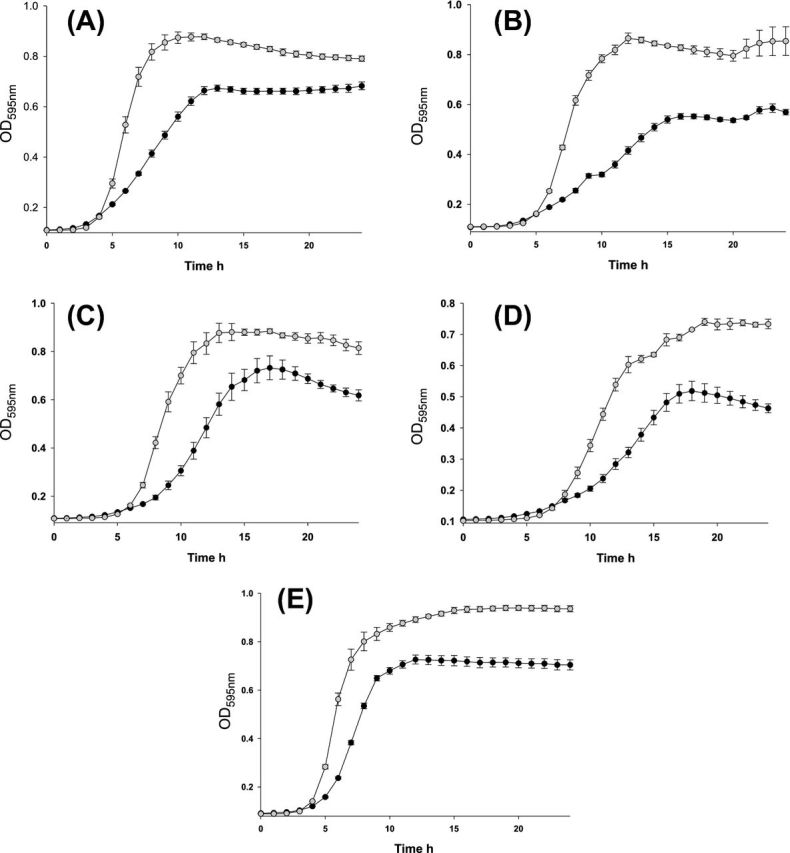
Confirmation of increased osmotolerance of AR1 (filled circle) in KCl and urea indicated By Biolog. (A) AR1 outgrows HB101:30 (filled circle) when grown aerobically in TSB 3% KCl. (B) AR1 shows a growth advantage over HB101:30 in TSB 4% w/v KCl. (C) Superior growth for AR1 compared to HB101:30 in TSB 5% w/v KCl. (D) A faster growth rate is noted for AR1 compared to HB101:30 in 6% w/v KCl. (E) A growth advantage in TSB 2% w/v urea is recorded for AR1. Optical density readings were taken at 595 nm. Error bars are standard deviations from the means of data from triplicate experiments. Significance was deemed to be a *P*-value ≤ 0.05.

Increased osmotolerance of AR1 in KCl and urea was indicated by Biolog phenotypic screens. Growth of the two bacteria in TSB containing various concentrations of KCl, namely 3%, 4%, 5% and 6% was observed over 24 h to confirm the Biolog indications (Fig. 3). AR1 exhibited better growth in all three concentrations with 6% KCl being the most notable difference with AR1 having a much sharper exponential phase than HB101:30 and remaining in stationary phase at 24 h while HB101:30 growth was declining (Fig. 3D). Growth advantages by AR1 can be seen in KCl 3% in Fig. 3A, 4% in Fig. 3B and 5% in Fig. 3C. A faster growth rate was also determined for AR1 in 2% urea where a sharper exponential phase and higher OD readings in the stationary phase were noted for AR1 compared to HB101:30 (Fig. 3E).

**Figure 4.**
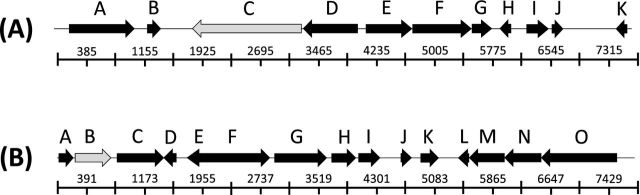
**(A)** KDp homologue and surrounding coding regions: (A) TnpA Transposase, (B) Mobile element protein/Transposase, (C) Osmosensitive K^+^ channel histidine kinase KdpD, (D) Two-component response regulator CreB, (E) Thiosulfate reductase cytochrome B subunit (membrane anchoring protein), (F) Sulfite oxidase and related enzymes (COG: 2041), (G) Hypothetical protein, (H) Hypothetical protein, (I) Mobile element protein/Transposase, (J) Hypothetical protein, (K) RepA1. **(B)** HN-S homologue and surrounding coding regions: (A) Haemolysin expression modulating protein, (B) DNA-binding protein H-NS, (C) Hypothetical protein (possible lipoprotein), (D) Hypothetical protein, (E) Hypothetical protein, (F) Resolvase, (G) Chromosome (plasmid) partitioning protein ParA/ Peptidyl-arginine deiminase, (H) Hypothetical protein/putative Methionine repressor-like protein, (I) Hypothetical protein/ Putative membrane protein, (J) Hypothetical protein/ Putative membrane protein, (K) Putative integral membrane protein, (L) Hypothetical protein, (M) DNA distortion protein, (N) Hypothetical protein/Putative replication initiation protein, (O) PI protein.

### Sequence analysis of plasmid DNA

Results from one tenth of a pyrosequencing titanium run were analysed further as follows. Using CAMERA 2.0's 454 Duplicate clustering tool, 1756 duplicate reads were removed, leaving 32 072 unique reads. These were analysed using the IMG/MER metagenomic pipeline (Markowitz *et al*. 2012). The reads were also assembled using CAMERA 2.0's meta-assembler, resulting in 793 contigs, totalling 578 654 bp. The contigs were uploaded to RAST, which predicted a total of 902 features; 14 RNAs and 888 protein coding genes. One of those predicted genes was 100% identical to H-NS found in multiple Enterobacteriaceae, and one was a histidine kinase containing a KdpD-like domain. IMG/MER analysis of unassembled reads predicted both H-NS and ‘K+ sensing histidine kinase KdpD’ genes, based on similarity of reads to domains in COGs 2916 and 2205 respectively. Both genes are involved in osmotolerance (Higgins *et al*. 1988; May *et al*. 1990; Lucht and Bremer 1991; Jung and Altendorf 2002; Laermann *et al*. 2013). The assembled contigs showed KdpD-like and the H-NS domain-containing gene neighbouring genes generally found in plasmids (Fig. 4A) and (Fig. 4B) respectively.

The contigs were subjected to BLAST analysis, and the alignments returned for each were screened for hits to known plasmids. These plasmid sequences were downloaded and aligned with all contigs that matched strongly (90% or more identity) to reconstruct the plasmids from the AR1 data. Where gaps between AR1 contigs were found in alignment with a known plasmid, the corresponding region of that plasmid was used as a query in a BLAST search of the AR1 data, which determined the best fit for smaller contigs in the alignment.

The sequences of five known *E. coli* plasmids were found to be 80%–100% complete within the plasmid data (Table 1). One of these, *E. coli* UMN026 plasmid p2ESCUM was found to contain the H-NS homologue.

**Table 1.** Most complete plasmids (≥80%) identified in sequence data.

Plasmid	Size	% of sequence
	bp	complete
*E. coli* strain EC29 plasmid pEC29-1	4082	100%
*E. coli* UMN026 plasmid p2ESCUM	33 809	≥80%
*E. coli* HUSEC41 plasmid pHUSEC41-3	7930	≥80%
*E. coli* plasmid ColE1	6600	≥80%

A number of other partial plasmid sequences were detected (<50%), one of which was *E. coli* ETEC H10407 plasmid p666 carrying the KdpD-like domain-containing gene mentioned above.

## DISCUSSION

The microbial community in the GIT is predominantly a viral and bacterial community, with the bacteria providing a myriad of beneficial activities from immune defence to vitamin production. The large intestine is the site where this community reaches its greatest numbers and density and faecal samples contain a mixture of transient and colonizing bacteria. Mobile elements are ubiquitous in ecosystems and are associated with community adaptations and the evolution of microbes to their environment. Here we investigated the distribution of plasmid DNA within the gut microbiota using an approach which relies upon exogenous matings to an *E. coli* host and describe the isolation of plasmid DNA that confers an ability to survive elevated osmolarity.

Gut microbes endure many stresses including osmotic stress. Osmoadaptation is a beneficial characteristic gut pathogens have expressed, allowing them to survive stressful osmotic environments associated with the disease state. Should this trait be plasmid borne it may potentially be transferred between pathogens and commensals during transient passage through the gut. Gut contents have been shown to have a salinity of ∼0.9%, similar to that of plasma serum (Fordtran *et al*. 1965), however, small areas of concentrated ions may gather in the lateral intercellular spaces and crypts leading to increased salt concentrations in these niches (Chatton and Spring 1995; Spring 1998) and changes in water content of the large intestine leads to osmotic fluctuations. Experiments by Licht and colleagues indicated that much of plasmid transfer occurs in the mucous layer covering the epithelial cells (Licht *et al*. 1999). The mucus layer has been found to be thickest in the colon (Atuma *et al*. 2001) and could make this a candidate location for plasmid transfer in the large intestine. Mucus is also a niche that commensal *E. coli* are believed to occupy (Chang *et al*. 2004), a fact which supports the use of the *E. coli* as the recipient in this study. The electrolyte content of the mucus layer is believed to be similar to that of serum or bile (Creeth 1978). The recipient strain used in these experiments was difficult to recover on agar containing 4% w/v NaCl, offering an opportunity to screen for plasmid-mediated osmoadaptation in faeces above that of sea water (3% w/v NaCl). Such tolerance may be of relevance in certain small niches of the gut, but also outside of the gut in food processing and other environmental niches.

Growth analysis providing confirmation of the superior growth by AR1 in salt and M9 minimal media was most striking as it displayed an advantage in high and low concentrations of salt, indicating AR1 has a mechanism to cope in low levels of NaCl. These results suggest an advantage for AR1 in sub-optimal as well as higher levels of NaCl and could indicate conditions where the acquired plasmids are most advantageous to the host.

In order to understand how our plasmids were modifying the bacterial host we used physiological profiling. The Biolog platform is a powerful means of assessing the phenotype of a bacterial strain. A recent study by Fabich and colleagues describes Biolog's pivotal role in the elucidation of a colonization advantage noted in an intestine adapted *E. coli* K12 mutant. Biolog is a platform that employs 96-well phenotypic microarrays to assess phenotypic variation in microbes, it measures metabolic activity rather than optical density. Using Biolog phenotypic screens Fabich and co-workers were able to identify carbon utilization as the basis of superior colonization of the gut by the mutant strain (Fabich *et al*. 2011). To our knowledge, the current study is the first report in which Biolog phenotypic screens have been used to explore plasmid-mediated phenotypes by comparing a plasmid-containing strain to an empty recipient. The Biolog system was used as an indicator of possible phenotypes worthy of further study in the plasmid-containing *E. coli* that otherwise may not have been studied. The method indicated many unexpected substrates in which AR1 exhibited a better metabolic output than HB101:30. The osmolyte screens reiterated the differences seen in AR1 when grown in NaCl compared to HB101:30. The screen also suggested a strikingly higher metabolic activity for AR1 in metal salts such as sodium and potassium chloride, compared to acid salts. An interesting result was AR1's metabolic activity in urea 2% which equalled that in NaCl at 5.5% concentration. The screens suggested that the presence of compatible solutes in media can also increase its metabolic capability. A handful of the findings were confirmed with automated growth curves, namely AR1's superior growth rate, in KCl 3%, 4%, 5% and 6% as well as its advantage in urea 2%. These growth comparisons of the empty recipient and the plasmid-containing AR1 provided evidence that the plasmid DNA in AR1 was contributing to salt tolerance. This may be an important asset to *E. coli* attempting to colonize the gut and even beyond the gut where KCl is a commonly used food preservative.

### Possible mechanisms of plasmid-mediated osmotolerance

Sequencing of total plasmid DNA from the isolated transconjugants AR1 lead us to conclude that a large amount (250 136 bp) of plasmid DNA seems to have been transferred during the conjugative experiments. Such a finding is not an anomaly as can be seen in the size of plasmids captured in previous mating studies (Drønen *et al*. 1998) and the recent works of Wang and co-workers (Wang *et al*. 2013).

Potassium homoeostasis was a very interesting functional prediction from RAST and IMG/MER. The first of two potassium homoeostasis related genes in the raw data by IMG/MER was a member of the osmosensitive high affinity potassium transport system Kdp, whose expression is under control of a two component regulatory system. This system is in addition to low affinity constitutively expressed transport systems such as TrKA and TrkD, and increased activity of the KdpD transport system is due to increased gene expression. It is the only known bacterial K^+^ transport system whose expression is rigidly controlled at the level of transcription (Epstein 2003). The KdpD-like domain-containing histidine kinase detected in the assembled reads by RAST was found to be surrounded by genes generally associated with plasmids suggesting its mobile nature within the gut mobile metagenome. Under potassium limitation or osmotic shock, the KdpD sensory kinase is responsible for activating the high affinity Kdp transport system. The responsivity of such a system to transcriptional control could create a situation where a plasmid borne sensory kinase of this kind, such as the homologue detected in this study, could exert effect on the transport system in the host. Under stressful conditions the plasmid borne kinase may facilitate increased expression of the signal for potassium transport and may have the potential to up-regulate the host response to osmotic shock. This homologue could be a candidate for further study as a plasmid-mediated mechanism for stress response in the healthy gut environment. Additionally, an H-NS homologue was identified in the data, the H-NS nucleoid protein exerts repression effects upstream of KdpD and senses K^+^ (Ueguchi and Mizuno 1993).

## CONCLUSION

The study indicates mobile spread of an osmotolerance phenotype to an *E. coli* recipient from the healthy gut and could have implications in the food industry where sodium and potassium chloride are used as preservatives. With the molecular mechanism of the trait unclear, further study of the KdpD*-*like domain-containing histidine kinase and H-NS homologue detected in the study may be warranted.
